# P04.19. T’ai chi as exercise among middle age and elderly Chinese in urban China

**DOI:** 10.1186/1472-6882-12-S1-P289

**Published:** 2012-06-12

**Authors:** G Birdee, H Cai, Y Xiang, G Yang, H Li, W Zheng, X Shu

**Affiliations:** 1 Vanderbilt University Medical Center, Nashville, USA; 2 Shanghai Cancer Institute, Shanghai, China

## Purpose

T’ai chi is a common form of mind-body practice used as a form of exercise in China. Limited data are available on the characteristics of t’ai chi practitioners in China and factors associated with t’ai chi use.

## Methods

We analyzed characteristics of and factors associated to t’ai chi practice among middle age and elder Chinese in Shanghai China utilizing baseline data from the Shanghai Women’s Health Study (n=74941, aged between 40-70 years) and Shanghai Men’s Health Study (n=61,491, aged 40-74 years). Validated questionnaires were administered in-person collecting data on physical activity including t’ai chi practice, sociodemographics, other health behaviors and chronic medical conditions. Logistic regression modeling was used to identify independent factors associated with the practice of t’ai chi among men and women.

## Results

T’ai chi is a common form of exercise in Shanghai, China among women (28%) and men (15%). A majority of adults that practiced t’ai chi used t’ai chi as their exclusive type of exercise. Women and men who practiced t’ai chi were more likely to be older, more educated, currently not working, and more likely to have chronic medical conditions including pulmonary, gastrointestinal, and cardiovascular diseases. Men who practiced t’ai chi were more likely to have cardiovascular diseases. T’ai chi activity was associated with other healthy behaviors including non-smoking, drinking tea, consuming ginseng, watching less TV, and participating in other forms of exercise.

## Conclusion

T’ai chi is the predominant form of exercise among middle age and elderly Chinese in urban China, particularly among those with older age and chronic medical diseases. Future research is needed to see if t’ai chi has similar or different benefits than conventional forms of exercise and physical activity.

